# Network loss following the 2016 Presidential Election among LGBTQ+ adults

**DOI:** 10.1007/s41109-022-00474-y

**Published:** 2022-06-17

**Authors:** Matthew Facciani, Tara McKay

**Affiliations:** Vanderbilt University, Nashville, USA

**Keywords:** Social networks, Political polarization, Alter loss, LGBTQ+, Kin ties

## Abstract

Growing levels of political polarization in the United States have been associated with political homogeneity in the personal networks of American adults. The 2016 Presidential Election in the United States was a polarizing event that may have caused further loss of connections to alters who had different politics. Kinship may protect against loss of politically different ties. Additionally, loss of ties with different political views may be particularly pronounced among LGBTQ+ people as they are more likely to be impacted by public policy decisions compared to their heterosexual counterparts. We analyzed two waves of the University of California, Berkeley Social Networks Study’s (UCNets) Main Sample and LGBTQ+ Oversample of older adults that occurred in 2015 and 2017, which provided an opportunity to assess alter loss after the 2016 Presidential Election. When evaluating all adults, we found that politically different alters were more likely to reflect kin ties than partner or friend ties. We also found that politically different kin are less likely to be dropped suggesting that kinship acts as a moderating effect of different political views on alter loss. LGBTQ+ respondents were more likely to drop kin alters with different political views than their cisgender heterosexual counterparts. We discuss the implications these results have for political polarization interventions as well as the social networks impact politics can have on LGBTQ+ individuals.

## Introduction

How does rising political polarization impact the networks of LGBTQ+ individuals in the United States? Democrats and Republicans have become more polarized over the last 40 years with growing ideological distance (Pew Research Center 2017) and mutual dislike for the political outgroup (Abramowitz and Webster 2018). This polarization is also associated with both Democrats and Republicans having politically homogenous networks (Survey Center on American Life 2021). Homogeneity is fueled in part by individuals reducing ties with people who have different political views; one survey estimates that 22% of Americans’ friendships ended because of disagreements over Donald Trump (Survey Center on American Life 2021). LGBTQ+ individuals reported significant psychological distress after the 2016 Presidential Election (Veldhuis et al. 2018), but less is known about how these polarizing times impacted their social networks and social support.

LGBTQ+ people, in particular, are more likely to identify as Democrats (Pew Research 2016) and are more likely to be politically engaged compared to heterosexual adults, making politics a particularly salient issue for this demographic (Vandermaas-Peeler et al. 2018). LGBTQ+ people may have been especially motivated to reduce contact with others who supported policies targeting LGBTQ+ and other minority groups following the election of President Donald Trump. Within hours of taking office, the Trump Administration removed all inclusive mentions of LGBTQ+ people and rights from official public facing materials and, by February, announced that the administration would no longer uphold protections for transgender students in the United States (O’Hara 2017). The magnitude of large-scale, multisite protests of President Trump and his administration’s policies in 2016 and 2017 were unprecedented (Andrews et al. 2018) and brought together coalitions motivated by a range of issues, including LGBTQ+ rights, reproductive rights, racial equality, immigration, and climate change (Fisher et al. 2017).

While politics could be a factor in network loss, other factors, like closeness and family ties, may help to preserve ties with individuals who have different politics (Fischer and Offer 2020). However, this may work differently for LGBTQ+ people who, prior to the 2016 Presidential Election, already had differently structured networks compared with their cisgender and heterosexual counterparts. Although there are few systematic studies of LGBTQ+ adults’ networks, especially those of older LGBTQ+ adults, existing work suggests that LGBTQ+ adults have higher representation of other LGBTQ+ people in their networks and older LGBTQ+ adults have less diverse networks compared with younger LGBTQ+ adults (Erosheva et al. 2016). Other social and structural issues, such as homophobia, rejection, and policy barriers, have left many LGBTQ+ people with fewer spouses, children, and other kin ties (Carpenter and Gates 2008; Jones 2017; Reczek and Bosley-Smith 2021). Some LGBTQ+ individuals adjust for lower kin support by constructing their own “chosen families” that are strong ties without a direct familial bond (Heaphy 2009; Hull and Ortyl 2018; Knauer 2016).

These opposing processes thus suggest two countervailing outcomes related to polarization and network homogenization in the aftermath of the 2016 Presidential Election: 1) LGBTQ+ people may have been particularly motivated to reduce ties to individuals with different politics in the aftermath of the Trump election to reduce conflictual, nonsupportive relationships, or 2) LGBTQ+ people may have already had more politically homogenous networks and developed strategies for maintaining ties to individuals with different political views, like kin, to the extent that these individuals appear in their networks. To rigorously examine how the networks of LGBTQ+ individuals changed relative to their cisgender and heterosexual counterparts before and after the 2016 Presidential Election, we use a before-and-after design in a large panel sample of adults that includes a substantial oversample of older LGBTQ+ identified people. The 2016 Presidential Election was a particularly polarizing event (Gentzkow 2016) and serves as a useful moment to expand research on how network ties to individuals with different political views change in response to political events and among LGBTQ+ adults, and understudied and politically engaged population. In the next sections, we review key findings in the literature on political polarization and social networks and LGBTQ+ people’s networks. We then present our data, which is uniquely suited to address how networks changed among older LGBTQ+ adults in this period. LGBTQ+ adults and older adults in particular have differently structured networks from their heterosexual counterparts. Our study aims to evaluate how LGBTQ+ individual’s networks were influenced by a major political event.

### Political polarization and social networks

The United States continues to become more polarized along partisan lines. Not only are Democrats and Republicans separating further on ideology (Pew Research 2017), but they are also growing in their dislike for each other (Abramowitz and Webster 2018; Finkel et al. 2020). Political polarization increases the strength and importance of political identities, which motivates partisans to process information that supports their own group while denigrating their outgroup (Van Bavel and Pereira 2018). Biased processing of political issues has been well-documented for Democrats and Republicans (Ditto et al. 2019), but such bias has grown to be quite broad. For example, Democrats and Republicans show a bias in hiring those who share their politics (Gift and Gift 2015), who they date (Easton and Holbein 2021), and often negatively stereotype each other (Deichert 2019). Studies have also shown how partisans will sometimes consider the political outgroup to be less than human (Martherus et al. 2021; Cassese 2021). A 2018 Pew Research Poll found that the majority of Democrats and Republicans believe that their differences go beyond policy as they cannot even agree on basic facts (Pew Research 2018). The broad bias of political polarization has been directly linked to the spread of misinformation (Van Bavel et al. 2020) and politization of health crises (Kushner Gadarian et al. 2020). Political scientists also express concern that such polarization threatens the foundation of our democracy (Levitsky and Ziblatt 2018).

Popular media reported on relationships ending during the 2016 Election (e.g., Filipovic 2016; Tavernise and Seelye 2016) and survey methods can help quantify the amount of network loss due to politics. A 2020 poll from the Survey Center on American Life found that 77% of Democrats and 75% of Republicans have politically homogenous social networks (Survey Center on American Life 2020). Social media also reveals significant political homogeneity as the average Facebook user was found to only have 23% of “friends” from the opposing political party (Bakshy et al. 2015). Not only do Democrats and Republicans avoid spending time with each other (Chen and Rohla 2018), they will even avoid conversing about nonpolitical topics such as sports or music (Settle and Carlson 2019). Lee (2021) found that close elections may even “close off” strong tied relationships because Americans are less likely to travel and have shorter Thanksgiving dinners following close elections. Thanksgiving dinners among politically diverse groups are also significantly shorter than those who agree on politics (Frimer and Skitka 2020). These studies are good indicators of a trend of political separation, but their designs do not directly test how political can change social networks overtime.

Social networks are an integral tool for evaluating the social outcomes of polarization as well as factors that contribute to further polarization. A 2020 Pew Research survey found both Trump and Biden supporters were less likely to have close friends with those who disagreed with their politics. This affects where people decide to live as well with 35% Democrats, and 50% of Republicans preferring to live in places with those who mostly share their politics (Pew Research 2014). Democrats are more than twice as likely to live in urban areas and Republicans are about twice as likely to live in rural areas across the United States (Mitchell 2018). This geographic and social distance further fuels political polarization. and could help explain why only 21% of Americans are in politically mixed marriages (Wang 2020).

Those with greater shared political agreement in their personal networks have stronger political beliefs (Bienenstock et al. 1990; Facciani and Brashears 2019) and the most extreme partisans are more likely to have politically homogenous networks on their social media (Boutyline and Willer 2017). Social influence does appear to impact beliefs directly as experiments show that having discussions with like-minded people increases belief strength (Keating et al. 2016) and respectful heterogenous discussions can decrease polarization (Levendusky and Stecula 2021a, b). Additionally, longitudinal network studies have found that people shift their ideological viewpoints to match their networks overtime (Lazer et al. 2010). Exposure different viewpoints does relate to polarization, but the type of relationships in the network matter. For example, exposure to opposing political information on social media does not reduce polarization (Bail et al. 2018) because following different Twitter accounts do not provide an opportunity for meaningful dialogue and reducing polarization through mutual understanding.

Facciani (2020) found that political heterogeneity within close networks was associated with reduced polarization. There was not an avoidance of discussion of politics with those who belonged to an opposing political group, but mutual respect was decreased in politically different network connections. Individuals are more likely to express political disagreement with those who they have strong ties with (Eveland et al. 2012). When people can learn more about the other side, they tend to become less polarized (Druckman et al. 2021; Levendusky and Stecula 2021a, b). Political disagreement can put stress on a relationship, but having a close connection appears to be critical for maintaining a politically different tie.

While researchers continue to uncover factors involved with political network composition, there is far less known about how political networks vary between different demographic groups. Race is a central political issue and could explain why White Biden supporters were twice as likely to have a Trump supporter in their network compared to Black Biden supporters (Pew Research 2020). Because marginalized groups are more likely to be impacted by current policies, political discussion may be especially relevant to them. The identities and human rights of LGBTQ+ individuals are often made into political issues, but there is a dearth of literature on how polarization shapes the networks of LGBTQ+ individuals.

### LGBTQ+ and political networks

The average LGBTQ+ individual is more likely to be liberal and support the Democratic party (Edelman 1992; Egan and Edelman 2008; Lewis et al. 2011; Perrella et al. 2012; Smith and Haider-Markel 2002), although bisexual and transgender individuals are somewhat less liberal than lesbian women and gay men (Jones 2021). LGBTQ+ individuals are also significantly more politically active than their heterosexual counterparts (Egan and Edelman 2008; Swank 2018, 2019; Turnbull-Dugarte and Townsley 2020; Vandermaas-Peeler et al. 2018).

This increased political engagement could be caused by how often LGBTQ+ individuals have their rights directly challenged from policies. When Donald Trump became President in 2016 and Republicans regained the White House, there were significant concerns that previous protections of LGBTQ+ individuals would be removed. This may explain the increased stress and decreased mental well-being LGBTQ+ individuals experienced after the 2016 Election (Gonzalez et al. 2018; Price et al. 2021). Interpersonal connections are key to reducing this psychological distress. Greater social support and community involvement have been associated with positive health outcomes among LGBTQ+ individuals (Budge et al. 2013; Dyar and London 2018; Kertzner et al. 2009; Ramirez-Valles et al. 2005). LGBTQ+ identity and political identity also appear to be mutually reinforcing as a 2020 poll by the Williams Institute found that 72% of LGB Democrats agree that it is important to be politically active in the LGBTQ+ community. Thus, LGBTQ+ individuals may be especially motivated to reduce connections with those who add stress and conflict to their lives as a result of holding different, less liberal political views.

Importantly, prior to the 2016 Presidential Election, LGBTQ+ adults already had differently structured networks. LGBTQ+ individuals have a higher proportion of LGBTQ+ individuals in their networks while also reporting less kin (Frost et al. 2016; Grossman et al. 2000; Shippy et al. 2004). The 2013 Windsor and 2015 Obergefell Supreme Court decisions have increased marriage rates among gay men and lesbian women (Carpenter et al. 2021; see also US Census 2018); however, LGBTQ+ individuals are still less likely to have spouses and committed partners compared to their heterosexual counterparts (Badgett et al. 2021). LGBTQ+ individuals who are married are also more likely to be white and more highly educated (Badgett et al. 2021) compared to unpartnered LGBTQ+ individuals. Additionally, policies that limit adoption for LGBTQ+ couples as well as large costs associated with assisted reproduction results in LGBTQ+ individuals being significantly less likely to have children (Carpenter and Gates 2008). For a historically marginalized group like LGBTQ+ people, a lack of spouse, child, and other kin ties, especially in the household, is due to social and structural barriers, including homophobia, rejection, and policies that have historically limited opportunities for family formation, like same-sex marriage bans and laws limiting adoption and access to reproductive technologies for LGBTQ+ families.

Some LGBTQ+ individuals adjust for lower kin support by constructing their own “chosen families” that are strong ties without a direct familial bond (Heaphy 2009; Hull and Ortyl 2018). LGBTQ+ people also engage in “conflict work” to maintain ties with challenging members of their families of origin (Reczek and Bosley-Smith 2021). Thus, the ties that remain in their networks through adulthood and into midlife and older ages may be particularly resilient kin and nonkin ties. While studies of network change in the general population demonstrate less turnover among older versus younger adults (Bowling et al. 1995; Conway et al. 2013; van Tilburg 1998), we expect this may be especially pronounced for minority groups like LGBTQ+ people who whose networks may reflect the effects of personal, social, and structural effects in more pronounced ways.

### The present study

The present study aims to describe the characteristics of network ties that hold different political views and investigates whether and how ties to individuals with different political views change after the 2016 Presidential Election. Drawing on network data collected before and after the 2016 Presidential Election, we also compare how network change before and after the election may differ for LGBTQ+ versus a random sample of cisgender and heterosexual adults. We first evaluate what types of relationships are most likely to contain political differences and predict politically network connections will be more likely to be kin or nonkin others because of political homogeneity within close networks (Facciani and Brashears 2019) and romantic partners (Easton and Holbein 2021). While individuals prefer political homogeneity, their kin may not share their political beliefs. We predict that politically different alters will be more likely to be kin compared to partners or friends.

**H1** Alters with different political views are most likely to be kin or nonkin others (e.g., acquaintances, neighbors, know at work) rather than spouses/partners or friends.

Social support is vital as network losses can reduce one’s ability to cope with stress (Gerstorf et al. 2011). However, the 2016 Election caused significant stress for many Americans, and this could motivate individuals to decrease ties to others with different political views (Gonzalez et al. 2018; Price et al. 2021). While political disagreement can cause stress in any relationship, we predict kin will be preserved despite having different political views for both LGBTQ+ and non- LGBTQ+ adults.

**H2** Kinship moderates the effect of different political views on alter loss for all adults.

**H2a** Kin alters with different political views are less likely to be dropped following the 2016 Presidential election.
**H2b** Nonkin alters with different political views are more likely to be dropped following the 2016 Presidential election.

LGBTQ+ people may have been especially motivated to reduce ties to individuals with different political views because of the salience of LGBTQ+ identities and other political issues during the 2016 election. However, they may also have already cut ties with politically different alters who reject them or developed coping strategies on how to maintain relationships with them (Reczek and Bosley-Smith 2021). Thus, we anticipate that there may be different patterns of loss and retention before and after the election for LGBTQ+ individuals compared to their heterosexual counterparts.

**H3** LGBTQ+ adults have different patterns of alter loss following the 2016 Presidential election compared with cisgender and heterosexual adults.

## Data and methods

We use data from the University of California, Berkeley Social Networks Study’s (UCNets) Main Sample and LGBTQ+ Oversample (Fischer and Offer 2020). The UCNets Main study collected data from a random address-based sample in the 6-county San Francisco Bay Area via face-to-face and online interviews. The UCNets sample includes two cohorts: 1) older adults aged 50 to 70 years old, and 2) young adults aged 21 to 30 years old at the time of wave 1 interview. Extended social media targeted advertising was also employed to collect data from a younger cohort. Retention in the UCNets Main study was high: 88% of respondents recruited in the first wave completed the second wave of the study. The main sample had a total of 1,018 respondents who completed both waves (134 of which identified as LGBTQ +).

The UCNets LGBTQ+ Oversample was obtained via convenience and venue-based sampling (such as Pride events and LGBTQ+ community centers) since sufficient LGBTQ+ samples are difficult to recruit using random sampling methods. The survey for the LGBTQ+ sample was administered entirely online and had a targeted recruitment of older adults only. The LGBTQ+ sample had a total of 307 respondents who completed both waves. The LGBTQ+ Oversample’s retention rate was 90% across all three waves. Both surveys were funded from the National Institute of Aging and reviewed by UC Berkeley’s and Vanderbilt University’s Institutional Review Board. The present study combined all LGBTQ+ identified individuals from both surveys into one sample to contrast with the heterosexual sample. This resulted in 884 heterosexual identified individuals and 441 LGBTQ+ identified individuals in our two main samples.

The UCNets Main study and LGBTQ+ Oversample both include three waves, but we focus on Waves 1 and 2 to observe network churn before and after the 2016 Presidential Election. The first wave was conducted from May 2015 to June 2016 and the second wave was conducted from February 2017 to June 2017 for the UC Nets Main Sample. In the LGBTQ+ Oversample, the first wave was conducted from October 2015 to June 2016 and the second wave was conducted from February 2017 to June 2017. Despite the slightly different collection times, both studies finished Wave 1 data collection before the 2016 Election results and began Wave 2 after Donald Trump was in office.

### Ego and alter characteristics

Our analyses rely on the identification of respondents by sexual orientation and gender identity. The UCNets Main study and the LGBTQ+ Oversample asked slightly different questions to assess sexual orientation and gender identity based on questions used in available studies as of 2014 and 2015. For the UCNets Main study, gender was asked as a male or female. For the UCNets LGBTQ+ Oversample, gender was asked as male, female, transgender male, transgender female. For this reason, we cannot differentiate transgender from cisgender adults in the Main sample and we cannot differentiate transgender from other gender diverse identities (e.g., nonbinary, gender nonconforming, gender queer) in either study.

For sexual orientation, the UCNets Main survey asked “How would you describe your sexual orientation?” with the responses (1)“Heterosexual or ‘straight’”, (2)“Homo-sexual or ‘gay’”, (3)“Something else”. Based on population data, we expect that individuals identifying in this category are most likely to identify as bisexual (Gates 2011). For the LGBTQ+ Oversample, sexual orientation was assessed using the two-step sequence “How would you describe your sexual orientation?”, with the responses (1)“Lesbian or gay”, (2)’Straight, that is not gay”, (3)“bisexual”, (4)“Something else”, or (5) “Don’t know”. Individuals selecting either “Something else” or “Don’t know” were asked a follow up question that presented additional identifications to assess their reasons for selecting either response. We have harmonized the variation in coding across the two instruments; however, we cannot disaggregate individuals identifying as bisexual from those identifying as “Something else” in the Main sample because of how the question was asked. For this reason, we aggregate data in the LGBTQ+ sample to match coding in the Main sample for bisexuals and others who identify as “Something else.” Beyond sexual orientation, participants were asked to report their sex, age, gender, and education level (college degree vs less than college degree). Participants also listed their political affiliation (Democrat, Republican, or Something else).

The respondents also answered a variety of networks questions to obtain their network composition and alter characteristics. The networks questions were derived from a variety of name-eliciting questions that is common in the social network literature (Laumann 1973; Fischer 1982; Marsden 2006). Respondents were asked to name the people to whom they were (1) married or partnered, (2) with whom they lived, (3) got together socially, (4) confided in, (5) from whom they sought critical advice, (6) received routine practical help, (7) could get significant emergency help, (8) whom they regularly helped, and (9) whom they found difficult. Respondents provided information on 17,886 unique alters, a majority (79.95%) of whom were observed in both waves.

From their list of alters, the respondent was asked to identify whether each name differed from the respondent in their political views (there is only a “yes” or “no” answer to this question because this is a check yes if they know for sure). Respondents were also asked to report specific descriptions of their alters including: subjective feeling of closeness, relationship (e.g. parent, child, friend, neighbor, coworker, etc.), and homophily on a set of demographic indicators (same race, same age, same gender, etc.). Previous research has shown that respondents are largely accurate when reporting the characteristics of their networks (Freeman et al. 1987; Freeman 1992; Perry et al. 2018), but respondents may also underestimate the amount of political disagreement in their networks (Goel et al. 2010).

Names of alters who were listed in Wave 1 but not listed in Wave 2 were categorized as “lost.” Other work with the UCNets Main sample has considered the variety of reasons that alters are “lost” over time, including mentions of “relationship break” or other disagreements, forgotten, or that they “drifted apart.” We conduct our main analyses using all alters who are lost rather than only those identified by the respondent as lost due to a politically motivated break in the relationship. This is because we anticipate that respondents may not openly or reliably identify different politics as a reason for no or less contact with any given alter.

### Analyses

First, we use descriptive and bivariate analyses to identify respondent and alter characteristics associated with political heterogeneity and alter loss at Wave 2 immediately after the 2016 Presidential Election. For both the LGBTQ+ and heterosexual respondents, we compare demographics (age, gender identity, race, education, and political affiliation), how many alters are politically different, how many alters are lost, and the relationships of the alters to the ego.

Next, we conduct a series of two-level logistic regressions with alters nested in waves predicting whether an alter has different political views and predicting loss at Wave 2. To test H1, we predict the likelihood that an alter was identified as having different political views as a function of a set of respondent characteristics (sexual orientation, gender, age, race/ethnicity, education), a set of alter characteristics (relationship, closeness, same race, same age), and survey design effects (older cohort, interview mode). To test whether kinship moderates the relationship between different politics and alter loss in H2, we predict the likelihood that an alter was not named in Wave 2 as a function of the interaction of alter relationship to respondent with alter political views and all the covariates in the model for H1. H3 proposes a three-way interaction between respondent sexual orientation/gender identity, alter relationship to respondent, and alter political views. This model predicts loss at Wave 2 like H2 and includes all of the covariates included in the models for H1 and H2.

## Results

Table 1 presents the sample characteristics for UCNets by sexual orientation and gender identity for all respondents present in Wave 1 and Wave 2 of the UCNets Main Study and the LGBTQ+ Oversample (about 10% of Wave 1 respondents did not complete Wave 2). LGBTQ+ respondents in UCNets do not significantly differ from heterosexual respondents on gender or education. However, LGBTQ+ respondents are significantly more likely to identify as white (χ^2^ = 15.20; *p* < 0.01). Due to the oversampling of older LGBTQ+ adults, LGBTQ+ identified respondents are also more likely to be older (*t* = 4.37; *p* < 0.01) and belong to the older age cohort (χ^2^ = 53.38; *p* < 0.01) compared with cisgender heterosexual respondents. LGBTQ+ respondents were also more likely to identify as a Democrat (*p* < 0.01), and less likely to identify as Republican (*p* < 0.01) or “something else” compared to cisgender heterosexual respondents (*F* = 21.94; *p* < 0.01). The majority (78.2%) of our respondents who identified as LGBTQ+ identified as either a gay man or lesbian woman.

Table 2 presents alter characteristics by respondent sexual orientation and gender identity. A plurality of alters are friends for both cisgender heterosexual respondents (50.9%) and LGBTQ+ identified respondents (61.8%). However, LGBTQ+ respondents were significantly less likely to list in their Wave 1 networks kin (*p* < 0.001), and more likely to list friends (*p* < 0.001) and nonkin others (*p* < 0.001). Nearly half (46.3%) of alters present in Wave 1 are lost at Wave 2 across the full sample. This proportion is lower for cisgender heterosexual respondents (45.2%) compared to LGBTQ+ identified respondents (48.4%; *p* < 0.001). LGBTQ+ identified respondents were also less likely to have alters with different political views in their networks (16.7% versus 23.5%; *p* < 0.001), more likely to have same age alters (63.5% versus 58.4%; *p* < 0.001), and less likely to report being very close emotionally to alters in their networks (52.4% versus 55.9%; *p* < 0.001) compared with cisgender heterosexual respondents.

In Table 3, we show patterns of alter retention and loss across alter characteristics for the overall sample, cisgender heterosexual respondents, and LGBTQ+ identified respondents. LGBTQ+ respondents were more likely to lose kin, friends, and nonkin others (*p* < 0.001) compared with cisgender heterosexual respondents. We also observe a lower proportion of alters with different political views lost to LGBTQ+ respondents compared with cisgender heterosexual respondents (15.1% versus 18.4%, *p* < 0.01).

In Table 4, we present the first analyses to directly test our hypotheses. H1 anticipates that alters with different political views are more likely to reflect certain kinds of relationships, especially kin relationships, where obligation binds the respondent to the alter despite differing political views, and nonkin others who are neither partners nor friends, where social relationships may be stickier (e.g., neighbors) and/or less under the control of the respondent (e.g., a coworker or service provider). Using a two-level (alters nested in respondents at wave 2) logistic regression model predicting whether the respondent has identified an alter as having different political views, we find that kin are more than two times more likely to be identified as having different political views compared to partners (OR = 2.185; 95% CI = 1.723–2.771]. Nonkin others have a positive odds ratio predicting different political views, however this is not statistically different from 1 (OR = 1.030; 95% CI = 0.781–1.357). This partially confirms H1, that alters with different politics are more likely to reflect kin ties than partner or friend ties. In this model, gay and lesbian respondents and more educated respondents were also markedly less likely to have alters with different political views (OR = 0.590; 95% CI = 0.435–0.801).

**Fig. 1:**
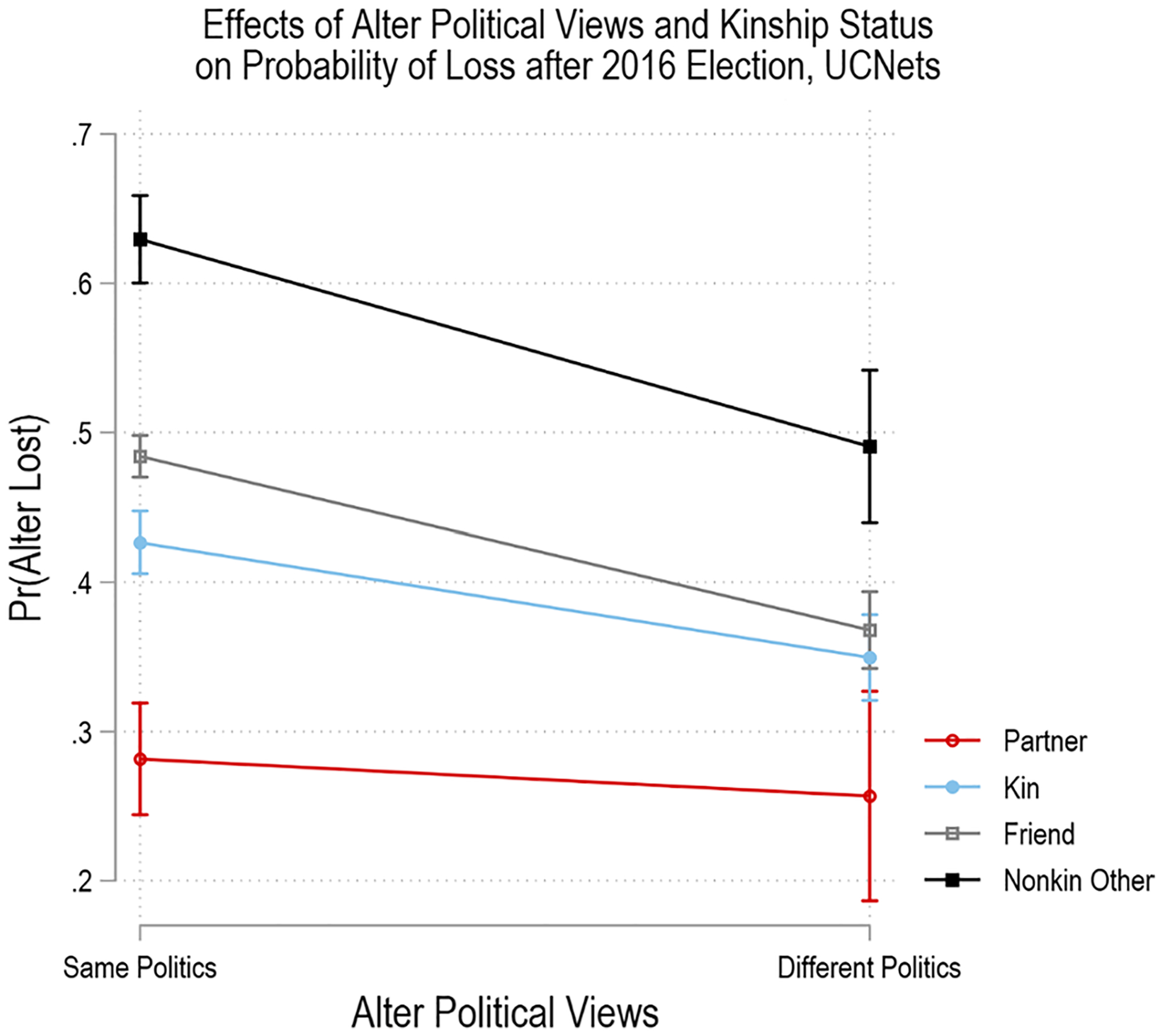
Effects of alter political views and kinship status on probability of loss after the 2016 Election, UCNets

Table 5 presents analyses examining H2, that kinship moderates the effect of different political views on alter loss for all adults in the UCNets sample. Here we allow the *alter has different politics* estimator to vary across *alter relationship to respondent*. We find main effects for each at *p* < 0.001. The two-way interaction effect is jointly significant at *p* < 0.05 and is primarily driven by differences in the effects of different politics for kin and friends in tests of simple effects of the alter has different politics estimator for each category of *alter relationship to respondent*. Figure 1 presents the pairwise comparisons of probability that an alter is lost at Wave 2 from the two-way interaction model testing H2. Here, we observe significant decreases in the probability of loss at Wave 2 for kin and friends when alters have different political views from the respondent. Compared to kin with the same political views, kin with different political views were about 35% less likely to be lost at Wave 2 for all respondents. Nonkin others are the most likely to be lost regardless of whether they have different political views in the overall sample. In H2b, we posit that nonkin alters with different political views will be more likely to be dropped at Wave 2 after the 2016 Presidential Election. We also observe that partners are retained at Wave 2 regardless of whether they have different political views. Overall, we find support for H2 (kinship moderates the effect of different political views on alter loss) and H2a (kin alters with different political views less likely to be dropped) specifically, but not strong support for H2b (nonkin alters with different political views are more likely to be dropped).

To test H3, that LGBTQ+ respondents will have different patterns of loss across alter relationship and alter political views, we conduct a three-way interaction model that allows the relationship between alter political views and alter relationship to respondent to vary across respondent LGBTQ+ identity (see Table 6). We find main effects of an alter having different political views (*p* < 0.001), of alter relation to respondent (jointly significant at *p* < 0.001), and of respondent LGBTQ+ identity (*p* < 0.05). The two- and three-way interaction effects are jointly significant at *p* < 0.001. This three-way interaction effect is driven primarily by a strong two-way interaction in the relationship between *alter relation to respondent* and *alter has different political views* for LGBTQ+ respondents. We observe simple effects of different political views significant at the *p* < 0.05 level for all relationship types except 1) partners among both cigender heterosexual and LGBTQ+ respondents and 2) kin among LGBTQ+ respondents.

**Fig. 2:**
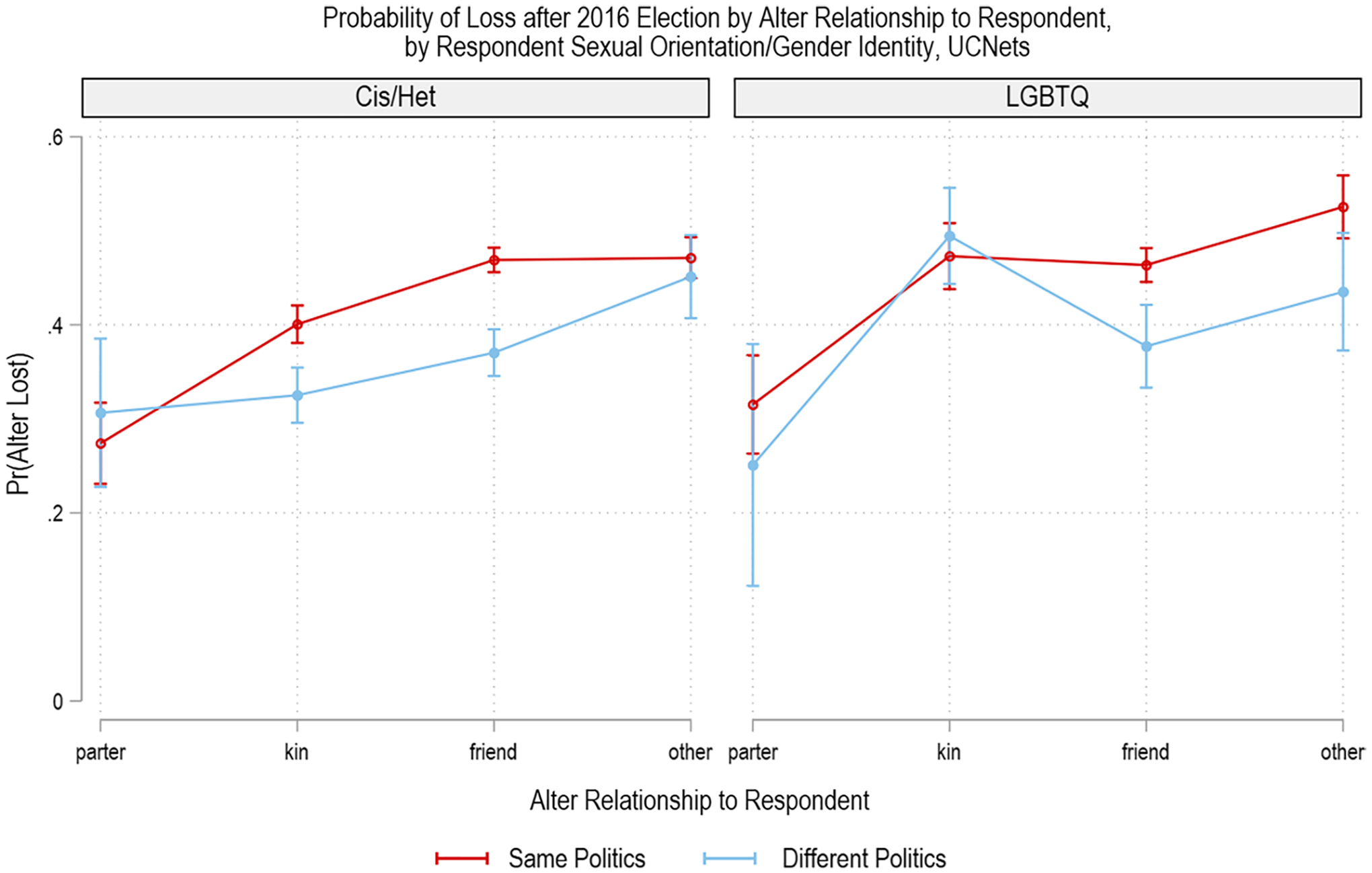
Probability of loss after 2016 election by alter relationship to respondent, by respondent sexual orientation/gender identity, UCNets

We visually present the predicted probabilities of alter loss with confidence intervals for the three-way interaction model for ease of interpretation in Fig. 2. Here, the differences in the effects of alter relationship to respondent clearly vary for kin from the left panel depicting cisgender heterosexual respondents compared with the right panel depicting LGBTQ+ respondents. For cisgender heterosexual respondents, kin and friends with different political views are significantly less likely to be lost after the 2016 Election compared with kin and friends who have similar politics, respectively. Partners of cisgender heterosexual respondents are least likely to be lost regardless of their political views, while nonkin are most likely to be lost regardless of their political views.

For LGBTQ+ respondents, we observe differences in the pattern of effects across alter relationship to respondent, with nonkin alters of LGBTQ+ respondents being highly susceptible to loss with or without different political views. Similarly, kin alters with different political views from LGBTQ+ respondents are not protected from loss and have elevated probabilities of loss relative to kin alters with different political views of cisgender heterosexual respondents. Thus, we find support for H3, which hypothesized that LGBTQ+ respondents may have different patterns of alter loss across relationship type when alters held different political views.

## Discussion

With growing polarization levels in the United States, we were interested in network loss among politically different alters before and after the 2016 Presidential Election. We analyzed the UC Nets Main Sample and LGBTQ+ Oversample longitudinal dataset to evaluate which alters are politically different, if kinship protects against alter loss, and if LGBTQ+ respondents are more likely to drop politically different alters. When evaluating all adults, we found that politically different alters were more likely to reflect kin ties than partner or friend ties. Furthermore, we found that politically different kin are less likely to be dropped suggesting that kinship acts as a moderating effect of different political views on alter loss. While politically different alters may be a source of strain on the relationship, it appears kinship still provides significant protection against alter loss for cisgender heterosexual adults. The protective properties of kin are consistent with other research (Fischer and Offer 2020). While there may be a threshold of difference in political views where kin are no longer protected from loss, measures in the UCNets survey do not allow us to gauge the *extent* of political difference between ego and alter.

When comparing cisgender heterosexual individuals and LGBTQ+ individuals, we found that LGBTQ+ people had a significantly lower proportion of politically different alters in their networks. LGBTQ+ individuals were also more likely to identify as Democrats and less likely to identify as Republicans, revealing higher levels of political homogeneity in their networks. LGBTQ+ respondents were more likely to list friends in their networks and less likely to report kin compared to heterosexual respondents. We also found that LGBTQ+ respondents reported different patterns of alter loss following the 2016 Presidential Election compared with cisgender heterosexual adults. Specifically, LGBTQ+ respondents were more likely to drop kin alters with different political views compared to their cisgender heterosexual counterparts. LGBTQ+ respondents being more likely to drop politically different kin alters may have been due to the salience of LGBTQ+ identities and other political issues during the 2016 election. The literature and prevailing mechanisms suggested competing processes whereby kin may have been both protected from loss and more likely to be among those that LGBTQ+ people cut ties with. For example, LGBTQ+ adults may have already cut ties with a significant amount of their politically different kin before the 2016 Presidential Election, and thus would have had fewer kin to lose. Alternatively, LGBTQ+ identities and rights have become increasingly salient, and this salience could result in greater effort to cut ties perceived as non supportive or hostile to LGBTQ and related issues. Our results suggest that LGBTQ+ individuals still had a nontrivial number of ties to politically different kin prior to the 2016 Election and that the political conflict contributed to some of these ties being severed. Although we expected to find larger differences for nonkin others, these relationships may be more resilient despite different political views because they are not easily dissolved or are more costly to dissolve for LGBTQ+ people (e.g., coworkers or neighbors with different political views).

Our results provide several important implications when considering the social problem of political polarization. We found that kinship acts as a buffer against dropping people from our networks who have different political views for cisgender heterosexual people but not for LGBTQ+ people. Research has shown that having civil discussions with members of the political outgroup can reduce misperceptions of the political outgroup and subsequently reduce political polarization (Levendusky and Stecula 2021a, b). While many Americans avoid political discussion, they are still more likely to share political opinions with friends and family (Cowan and Baldassarri 2018). Organizations such as Braver Angels can help provide resources for those interested in navigating these challenging conversations (Baron et al. 2021). However, the results of these depolarization efforts may not properly account for the additional political conflict that marginalized groups experience. Further work is needed to incorporate how depolarization efforts can occur with different marginalized groups and if different techniques are required.

Our study also reveals how LGBTQ+ identity influences political networks. LGBTQ+ individuals frequently have their rights up for political debate, which increases stress and decreases mental well-being (Gonzalez et al. 2018; Price et al. 2021). Due to the added strain of politics, it is understandable why LGBTQ+ individuals are more likely to drop politically different kin alters from their networks. Therefore, it may be easier for cisgender heterosexual individuals to have civil discourse with politically different alters in their networks in order to reduce political polarization. Finally, since LGBTQ+ individuals are often less likely to have spouses and partners compared to their heterosexual counterparts, it is especially important to consider how they may lose network connections and social support due to political polarization.

### Limitations and future directions

Our LGBTQ+ sample was predominantly composed of gay men and lesbian women. The weaker effects for politically different alter loss for transgender/gender-nonconforming, bisexual men, and bisexual women individuals may be due to their small sample size. We will need a larger sample size to test if these results hold or if there is potentially something different in alter loss between various groups within the LGBTQ+ population. Furthermore, our samples were predominantly white and highly educated so we were not able to conduct meaningful analyses of race or class in our study. Another limitation of our study is that the data was collected entirely around the San Francisco Bay Area. This generated a particularly politically homogenous sample. However, it is important to note that finding any effects for politically different alter loss with such a homogenous sample suggests that even stronger effects could be found with more opportunities for political diversity.

The UCNets study did not ask questions specifically related to political ideology or attitudes towards LGBTQ+ policy. Future research could assess how politically different alter loss may influence political belief as well. A potential pathway between LGBTQ+ identity, network churn, and political belief may exist for future researchers to observe. Additionally, this study only has data from the respondents so the network churn of the alters listed is unknown. A future study could assess if there is agreement between individuals who drop alters and if those same individuals were dropped according to the alter listed. It is possible that respondents may drop individuals from their network, but those same individuals may not report dropping the respondent from their network. This dataset also did not have an adequate measure of network density so a future study could investigate how alter’s connections to other alter’s influences their likelihood of being dropped as well. Finally, a qualitative study following up with LGBTQ+ individuals who drop politically different alters may provide greater insight on why these ties were dropped and how the relationship was maintained before alter loss occurred.

## Conclusion

During high levels of political polarization, it is important to evaluate how political disagreement can impact networks and especially the networks of LGBTQ+ individuals who are directly influenced from various policies. We find that kinship protects against politically different alter loss for heterosexual cisgender individuals, but LGBTQ+ individuals were more likely to drop kin with different political views compared to their cisgender heterosexual counterparts. Thus, LGBTQ+ identity is an important characteristic to consider as networks change during these polarized times.

## Figures and Tables

**Table 1:** Respondent characteristics, by respondent sexual orientation, UCNets

	Total		Respondent sexual orientation/gender identity				*p*
			Cisgender heterosexual		LGBTQ+		
	No	%	No	%	No	%	
Female	783	59.0	590	66.7	193	43.7	***
Age (Mean/SD)	50.69	16.81	49.3	18.02	53.5	13.73	***
BA or more	975	76.4	656	76.8	319	76.1	
Non-Hispanic White	888	67.0	561	63.4	327	74.1	***
Political Identity							
Democrat	947	73.3	591	68.5	356	83.5	***
Republican	86	6.6	80	9.2	6	1.4	***
Something Else	255	19.7	191	22.1	64	15.0	***
Sexual Orientation/Gender Identity							
Gay Man	229	17.2	–	–	229	51.9	
Lesbian Woman	116	8.7	–	–	116	26.3	
Bisexual Man	13	0.9	–	–	13	2.9	
Bisexual Woman	77	5.8	–	–	77	17.4	
Transgender	6	0.4			6	1.3	

Excludes respondents who were lost to follow up at Wave 2.

* *p* < .05,

** *p* < .01,

*** *p* < .001

**Table 2:** Alter characteristics relative to respondent, by respondent sexual orientation/gender identity, UCNets

	Cisgender heterosexual respondent		LGBTQ respondent		*p*-value	Total	
	No	%	No	%		No	%
Alter present at Wave 1	8,585	100.0	4,289	100.0		12,874	100.0
Alter lost at Wave 2	3,877	45.2	2,078	48.4	***	5,955	46.3
Alter relation to ego							
Partner	535	6.2	304	7.1		839	6.5
Kin	2,613	30.4	762	17.8	***	3,375	26.2
Friend	4,373	50.9	2,649	61.8	***	7,022	54.5
Other	1,064	12.4	574	13.4		1,638	12.7
Alter different politics	2,017	23.5	716	16.7	***	2,733	21.2
Alter same race/ethnicity	6,530	76.1	3,212	74.9		9,742	75.7
Alter same age	5,009	58.4	2,724	63.5	***	7,733	60.1
Ego very close emotionally to Alter	4,794	55.9	2,247	52.4	***	7,041	54.7

Excludes networks of respondents who were lost to follow up at Wave 2.

* *p* < .05,

** *p* < .01,

*** *p* < .001

**Table 3:** Characteristics of alters who were lost at Wave 2, by respondent sexual orientation/gender identity, UCNets

Overall	Alters Lost at Wave 2								*p*-value
	Overall		Total		Cisgender heterosexual		LGBTQ		
	No	%	No	%	No	%	No	%	
Total	5,955	46.3	12,874	100.0	3,877	45.2	2,078	48.4	
Alter relation to ego									
Partner	123	2.1	912	100.0	70	1.8	53	2.6	
Kin	1,139	19.1	3,662	100.0	818	21.1	321	15.5	***
Friend	3,426	57.5	8,204	100.0	2,149	55.4	1,277	61.5	***
Other	1,267	21.3	2,013	100.0	840	21.7	427	20.6	
Alter different politics	1,026	17.3	2,733	100.0	713	18.4	313	15.1	***
Alter same race/ethnicity	4,087	68.7	11,310	100.0	2,610	67.5	1,477	71.1	**
Alter same age	3,371	56.7	8,903	100.0	2,153	55.7	1,218	58.6	*
Ego very close emotionally to alter	1,851	31.1	8,026	100.0	1,188	30.7	663	31.9	

Excludes respondents who were lost to follow up at Wave 2.

* *p* < .05,

** *p* < .01,

*** *p* < .001

**Table 4:** Estimates of the likelihood that alters hold different political views than the respondent, UCNets

	Alter has different political views	
	Odds ratio	95% confidence interval
Alter relation to R		
Partner	1	[1,1]
Kin	2.185***	[1.723,2.771]
Friend	0.947	[0.755,1.188]
Other	1.030	[0.781,1.357]
R sexual orientation		
Heterosexual	1	[1,1]
Gay/Lesbian	0.590***	[0.435,0.801]
Bisexual/something else	0.929	[0.601,1.437]
R Gender		
Male	1	[1,1]
Female	0.951	[0.750,1.207]
Transgender	0.975	[0.249,3.811]
R Age	1.011	[0.989,1.033]
R Older Cohort	0.497 +	[0.225,1.100]
R Person of Color	0.891	[0.695,1.143]
R College Degree or more	0.658**	[0.505,0.857]
R Very Close to Alter	0.955	[0.846,1.077]
Alter is same race as R	1.072	[0.926,1.241]
Alter is same age as R	1.085	[0.962,1.224]
Network Size	0.967*	[0.941,0.993]
Interview Mode Effects		
Face-to-Face	1	[1,1]
Online	0.838	[0.647,1.084]
Variance at the Respondent Level	12.90***	[8.896,18.70]
Alters (N)	12,861	
Respondents (N)	1,227	

Odds ratios are exponentiated logistic regression coefficients.

+ *p* < 0.1,

* *p* < 0.05,

** *p* < 0.01,

*** *p* < .001

**Table 5:** Estimates of the likelihood of alter loss at Wave 2 by political views and relationship to respondent, UCNets

	Alter Lost at Wave 2			
	Model I (main effects)		Model II (2-way interaction)	
	Odds ratio	95% confidence interval	Odds ratio	95% confidence interval
Alter different politics	0.569***	[0.508,0.638]	0.649***	[0.538,0.783]
Alter relation to R				
Partner	0.445***	[0.353,0.560]	0.429***	[0.332,0.556]
Kin	1	[1,1]	1	[1,1]
Friend	1.298***	[1.156,1.457]	1.365***	[1.199,1.554]
Other	2.764***	[2.336,3.270]	3.005***	[2.480,3.641]
Alter different politics X Alter relationship to R				
Partner with different politics	–	–	1.310	[0.755,2.273]
Kin with different politics	–	–	1	[1,1]
Friend with different politics	–	–	0.815 +	[0.641,1.036]
Other with different politics	–	–	0.725 +	[0.507,1.037]
R Sexual Orientation				
Heterosexual	1	[1,1]	1	[1,1]
Gay/Lesbian	0.960	[0.819,1.126]	0.957	[0.816,1.123]
Bisexual/something else	0.969	[0.770,1.219]	0.966	[0.768,1.215]
R Gender				
Male	1	[1,1]	1	[1,1]
Female	0.925	[0.815,1.050]	0.926	[0.816,1.051]
Transgender	1.520	[0.738,3.131]	1.519	[0.737,3.130]
R Age	0.992	[0.980,1.003]	0.992	[0.981,1.003]
R Older Cohort	1.067	[0.703,1.620]	1.067	[0.703,1.619]
R Person of Color	1.051	[0.920,1.201]	1.051	[0.920,1.201]
R College Degree or more	1.047	[0.906,1.211]	1.046	[0.904,1.209]
R Very Close to Alter	0.155***	[0.141,0.171]	0.155***	[0.141,0.172]
Alter is same race as R	0.621***	[0.554,0.695]	0.621***	[0.554,0.695]
Alter is same age as R	0.715***	[0.649,0.788]	0.716***	[0.650,0.789]
Network Size	0.933***	[0.919,0.947]	0.933***	[0.919,0.947]
Interview Mode Effects				
Face-to-Face	1	[1,1]	1	[1,1]
Online	1.199*	[1.042,1.379]	1.199*	[1.043,1.379]
Variance at the Respondent Level	1.556***	[1.419,1.706]	1.555***	[1.418,1.704]
Alters (N)	12,861		12,861	
Respondents (N)	1,226		1,226	

Odds ratios are exponentiated logistic regression coefficients.

+ *p* < 0.1,

* *p* < 0.05,

** *p* < 0.01,

*** *p* < .001

**Table 6:** Estimates of the likelihood of alter loss at Wave 2 with 3-way interaction by political views, relationship to respondent, and respondent sexual orientation, UCNets

	Alter Lost at Wave 2	
	Model III (3-way interaction)	
	Odds ratio	95% confidence interval
Alter has different politics	0.564***	[0.452,0.704]
Alter relation to R		
Partner	0.420***	[0.300,0.588]
Kin	1	[1,1]
Friend	1.568***	[1.348,1.823]
Other	3.594***	[2.845,4.540]
Alter different politics X Alter relationship to R		
Partner with different politics	1.762 +	[0.918,3.380]
Kin with different politics	1	[1,1]
Friend with different politics	0.916	[0.690,1.216]
Other with different politics	0.919	[0.590,1.433]
LGBTQ+ Respondent	1.346*	[1.039,1.743]
Alter different politics X LGBTQ+ Respondent	1.589*	[1.039,2.431]
Alter relationship to R X LGBTQ+ Respondent		
Partner of LGBTQ+ Respondent	0.909	[0.541,1.530]
Kin of LGBTQ+ Respondent	1	[1,1]
Friend of LGBTQ+ Respondent	0.625***	[0.478,0.818]
Other of LGBTQ+ Respondent	0.552**	[0.372,0.820]
Alter different politics X Alter relationship to R X LGBTQ+ Respondent		
Partner with diff. politics of LGBTQ+ Respondent	0.373*	[0.141,0.987]
Kin with diff. politics of LGBTQ+ Respondent	1	[1,1]
Friend with diff. politics of LGBTQ+ Respondent	0.667 +	[0.445,1.000]
Other with diff. politics of LGBTQ+ Respondent	0.458**	[0.278,0.754]
R Gender		
Male	1	[1,1]
Female	0.987	[0.917,1.061]
Transgender	1.655*	[1.074,2.549]
R Age	0.993*	[0.986,1.000]
R Older Cohort	1.261 +	[0.985,1.614]
R Person of Color	1.041	[0.962,1.127]
R College Degree or more	1.077 +	[0.987,1.175]
R Very Close to Alter	0.330***	[0.309,0.352]
Alter is same race as R	0.776***	[0.723,0.834]
Alter is same age as R	0.806***	[0.757,0.859]
Network Size	0.930***	[0.922,0.938]
Interview Mode Effects		
Face-to-Face	1	[1,1]
Online	1.202**	[1.046,1.381]
Variance at the Respondent Level	1.549***	[1.414,1.698]
Alters (N)	12,861	
Respondents (N)	1,227	

Odds ratios are exponentiated logistic regression coefficients.

+ *p* < 0.1,

* *p* < 0.05,

** *p* < 0.01,

*** *p* < .001

## Data Availability

Data for the UCNets Main Sample and LGBTQ+ Oversample are available at https://ucnets.berkeley.edu/researcher-resources/data-documentation/.

## Abbreviation

LGBTQ+: Lesbian, gay, bisexual, transgender, queer, and other gender & sexual minority individuals
